# Proteomic Analysis of Human Brown Adipose Tissue Reveals Utilization of Coupled and Uncoupled Energy Expenditure Pathways

**DOI:** 10.1038/srep30030

**Published:** 2016-07-15

**Authors:** Sebastian Müller, Miroslav Balaz, Patrik Stefanicka, Lukas Varga, Ez-Zoubir Amri, Jozef Ukropec, Bernd Wollscheid, Christian Wolfrum

**Affiliations:** 1 Institute of Food, Nutrition and Health, ETH Zurich, Schwerzenbach, Switzerland; 2 Institute of Molecular Systems Biology, ETH Zurich, Zurich, Switzerland; 3 Life Science Zurich Graduate School, Molecular Life Sciences Program, Zurich, Switzerland; 4 Department of Otorhinolaryngology – Head and Neck Surgery, Faculty of Medicine and University Hospital, Comenius University, Bratislava, Slovakia; 5 Institute of Experimental Endocrinology, Biomedical Research Center at the Slovak Academy of Sciences, Bratislava, Slovakia; 6 UMR 7277, Centre National de la Recherche Scientifique, U1091, Institut National de la Santé et de la Recherche Médicale, Institute of Biology Valrose, University Nice Sophia Antipolis, Nice, France

## Abstract

Human brown adipose tissue (BAT) has become an attractive target to combat the current epidemical spread of obesity and its associated co-morbidities. Currently, information on its functional role is primarily derived from rodent studies. Here, we present the first comparative proteotype analysis of primary human brown adipose tissue versus adjacent white adipose tissue, which reveals significant quantitative differences in protein abundances and in turn differential functional capabilities. The majority of the 318 proteins with increased abundance in BAT are associated with mitochondrial metabolism and confirm the increased oxidative capacity. In addition to uncoupling protein 1 (UCP1), the main functional effector for uncoupled respiration, we also detected the mitochondrial creatine kinases (CKMT1A/B, CKMT2), as effective modulators of ATP synthase coupled respiration, to be exclusively expressed in BAT. The abundant expression and utilization of both energy expenditure pathways in parallel highlights the complex functional involvement of BAT in human physiology.

White adipocytes store energy to release it back to the body when needed, while brown adipocytes primarily convert energy to heat. Brown adipose tissue (BAT) is traditionally characterized by the unique expression of uncoupling protein 1 (UCP1), which is believed to be the major functional component, and by a distinct multilocular morphological appearance^1^,^2^. In man, the so called “classical” brown adipose tissue depot localized in the interscapular region, is only present in infants. Recently, however, it has been reported that functionally active brown adipocytes can be found interspersed in white adipose tissue depots in adults^3^,^4^,^5^,^6^,^7^. Moreover, epidemiological studies have demonstrated an inverse correlation between the presence or amount of brown adipose tissue and body weight as well as obesity associated co-morbidities^8^,^9^,^10^, stimulating a surge of research in BAT biology. Due to the limited amount of primary human material available, research on BAT heavily relies on transcriptome analysis^11^,^12^,^13^,^14^,^15^ or analyses of human adipose tissue derived, *in vitro* differentiated cells^16^,^17^,^18^. In contrast, information on the proteotype, the steady state protein abundance levels in primary brown adipose tissue of humans, is virtually non-existent. We present the first comprehensive proteotype analysis directly comparing human brown and adjacent subcutaneous white adipose tissues, identifying the increased abundance of 318 proteins in brown compared to white. These differences reflect not only the increased oxidative/thermogenic mitochondrial capacity, but also reveal brown fat’s exclusive expression of the mitochondrial creatine kinases CK-MT1A/B and CK-MT2. Interestingly, the coupled respiratory machinery consisting of the ADP/ATP-translocase, phosphate transporter and F1FO-ATPsynthase is also increased in abundance in the brown adipose tissue patient samples and we here demonstrate its functional importance to human brown adipocytes.

## Results

**Figure 1:**
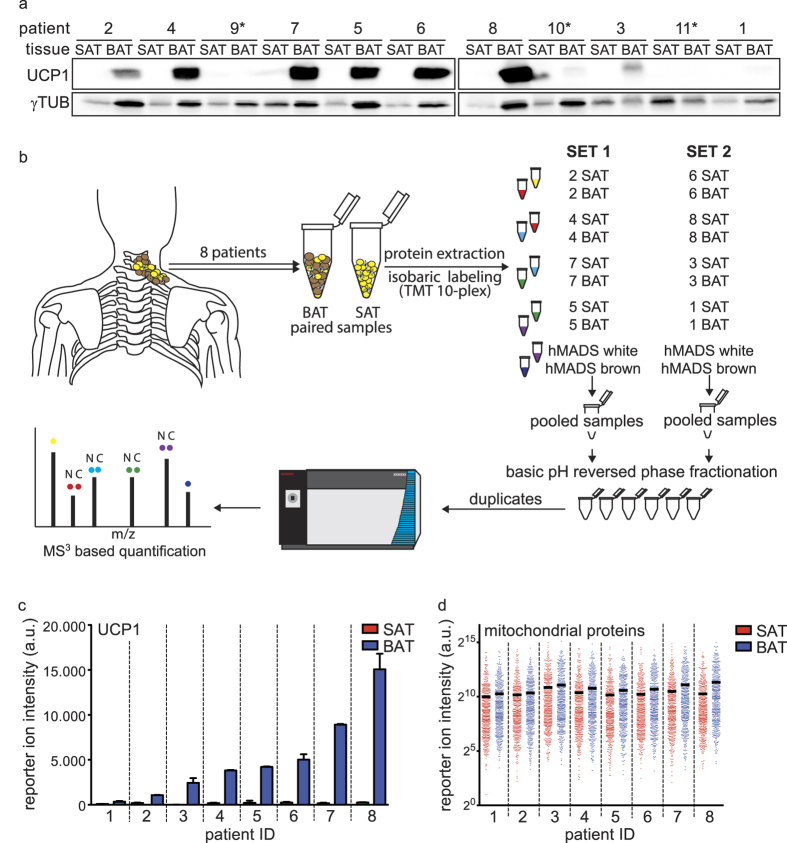
Initial characterization and set-up of the proteomic workflow for the analysis of paired SAT and BAT samples. (**a**) Western blot for UCP1, as BAT reference protein, and γ-Tubulin, as loading control to indicate uneven protein amounts due to different sample compositions, of paired SAT and BAT patient samples. Patients marked with an asterisk (*) were excluded from further analysis. (**b**) Overview of the quantitative proteomic work-flow. (**c**) Summed-up reporter ion intensities for UCP1 in the proteomic data. Error-bars represent the standard-deviation of the technical replicates (n = 2). (**d**) Summed-up reporter ion intensities of the 515 mitochondrial proteins across the paired patient samples. The median abundance level is indicated by the horizontal line.

To study the protein composition of primary human brown adipose tissue, we collected paired subcutaneous white (SAT) and deep neck brown adipose tissue (BAT) samples from a cohort of patients undergoing neck surgery. Analyzing the expression of UCP1 in paired samples from eleven patients (Supplementary Table S1), we detected varying levels, reflecting the biological heterogeneity between patients (see Fig. 1a). Based on this analysis, patients 9, 10, and 11 were excluded from further analysis due to the lack of detectable UCP1 expression in the BAT samples. To further elucidate the quantitative proteotype differences in-between white and brown adipose tissue, we applied an isobaric peptide labeling strategy followed by pre-separation and tandem mass spectrometric analysis of the samples (see Fig. 1b). *In vitro* differentiated hMADS cells were included in the analysis, as an established cell line model to study human brown and white mature adipocytes^19^. In total, we identified with high confidence 3488 proteins from limited amounts of surgically removed primary tissue, of which 2519 proteins could be consistently quantified across all samples (Supplementary Table S2).

Initial data analysis focused on specific sets of reference proteins to ensure high quality and comparability between the individual patient samples. As blood contaminations are unavoidable in the preparation of human whole tissue biopsies, we analyzed the abundance of the serum markers Albumin, Transferrin and Apolipoprotein B100 as well as the erythrocyte-specific proteins Hemoglobin Beta and Spectrin. We detected all serum markers evenly distributed between the paired patient samples (see Supplementary Fig. S1) and even though erythrocyte-specific proteins vary between the individual sample pairs, there was no bias towards either white or brown adipose tissue (see Supplementary Fig. S1). Therefore, we concluded that blood derived contaminants do not interfere systematically with our downstream analysis. Additionally, the abundance of common reference proteins such as Actin, HSP90, Tubulin and RPLP0 (see Supplementary Fig. S1) was comparable between SAT and BAT pairs, with only patient 2 and 5 slightly skewed towards the BAT sample. To validate our quantification strategy, we further compared the signal intensities for UCP1 obtained in the mass spectrometry based analysis with the levels obtained by western blot analysis and found clear correlation (see Fig. 1c). In agreement with the higher mitochondrial oxidative capacity of brown adipocytes^1^, we observed an increased median abundance of the 515 mitochondrial proteins (localization retrieved from MitoCarta 2.0^ ^20), quantified in our study. Moreover, this increase in mitochondrial protein abundance correlated well with the degree of UCP1 expression in our patient cohort (see Fig. 1d). This global increase in abundance of the mitochondrial proteins, however, is accompanied by a varied extent of enrichment of the individual proteins (see Supplementary Table S2).

### Specific proteins enriched in BAT samples are revealed by unsupervised clustering

**Figure 2:**
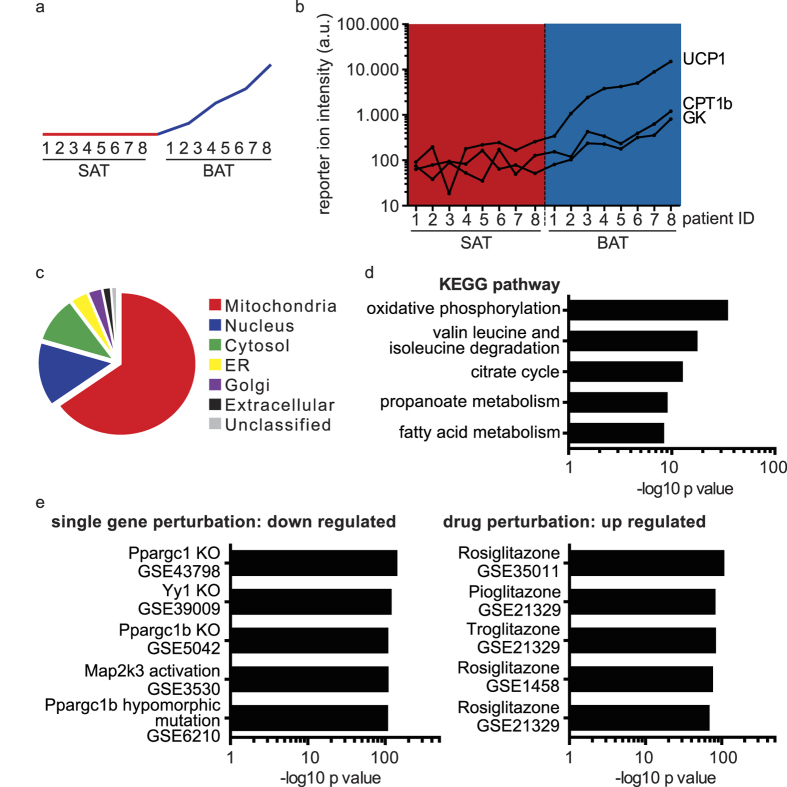
Unsupervised clustering reveals a set of proteins to be consistently upregulated in BAT samples. (**a**) Theoretical depiction of the abundance levels of the proteins found in the cluster upregulated in BAT. (**b**) Abundance levels of BAT markers UCP1, GK and CPT1β across the SAT and BAT patient samples in the proteomic study, presented as summed up reporter ion intensities. (**c**) Gene ontology cellular component analysis. (**d**) KEGG pathway enrichment analysis. (**e**) Gene-set enrichment analysis in publicly available transcriptome datasets.

To assess specific proteomic differences between human white and brown adipose tissue, we performed an unsupervised k-means clustering of the patient data. This unbiased approach revealed a cluster of proteins consistently higher in abundance in the brown adipose tissue samples and correlating with UCP1 abundance (see Fig. 2a). Among the 318 regulated proteins are classical brown adipose tissue markers such as UCP1, Glycerol Kinase (GK) and Carnitine O-palmitoyltransferase 1 beta (CPT1b) (see Fig. 2b). Furthermore, gene ontology (GO) analysis revealed the majority of proteins to be localized in mitochondria and associated with mitochondrial function (see Fig. 2c). Subsequent pathway analysis in the KEGG database^21^ revealed a significant enrichment in oxidative phosphorylation and other mitochondrial metabolic pathways (see Fig. 2d). We performed a gene-set enrichment analysis using publicly available datasets to explore the physiological relevance of this set of proteins. Thereby we found a significant association with genes negatively regulated by PGC-1α knock out and positively regulated by PPARγ agonist treatment in rodent studies (see Fig. 2e), confirming the functional importance of this protein cluster for brown adipose tissue biology. Collectively, our protein abundance profile demonstrates that next to a general difference in mitochondrial content, a specific set of proteins is differentially regulated in white and brown adipose tissue. This includes proteins with diverse functions, e.g. transferrin receptor (TFRC) for iron import, the lysosomal protease cathepsin d (CTSD) and lipase acid ceramidase (ASAH1) as well as prostaglandin E synthase 2 (PTGES2) involved in lipid metabolism (see Supplementary Table S2).

In the BAT biopsies brown adipocytes are interspersed in white adipose tissue and the contribution of white adipocytes to these biopsies cannot be estimated, due to the lack of a specific marker. Thus with our quantification scheme, we only detected single proteins enriched in SAT compared to BAT biopsies and not a clearly defined cluster (see Supplementary Table S2).

### Proteins of the mitochondrial interactosome are enriched in BAT compared to the SAT samples

**Figure 3:**
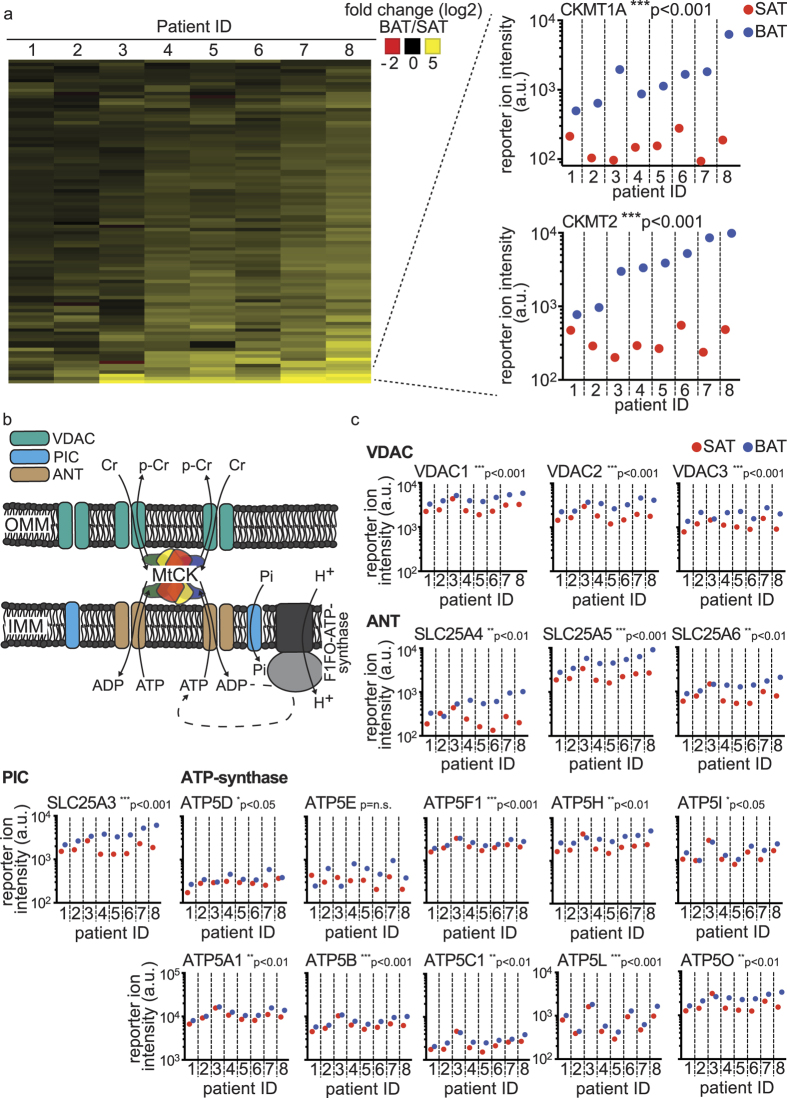
Mitochondrial creatine kinases and components of the mitochondrial interactosome are significantly enriched in BAT compared to the SAT samples. (**a**) Heat-map of the fold changes (log2) between the paired SAT and BAT summed up protein intensities of the top 100 proteins enriched in the BAT across the patient samples. Detailed view of the summed up protein intensities of the mitochondrial creatine kinases CK-MT1A/B and CK-MT2.(**b**) Depiction of the mitochondrial interactosome, as described in Timohhina *et al*.^23^. (**c**) Summed up reporter ion intensities across the SAT and BAT patient sample pairs for the ANT, VDAC, PiC and the ATP synthase protein complexes. Data was analyzed by ratio paired t testing of the SAT and BAT sample pairs (n = 8); *p < 0.05; **p < 0.01; ***p < 0.001. OMM = outer mitochondrial membrane; IMM = inner mitochondrial membrane; MtCK = mitochondrial creatine kinase complex; VDAC = voltage-dependent anion channel; PiC = phosphate carrier; Pi = phosphate; ANT = adenine nucleotide translocator.

Mapping the top 100 proteins in the brown adipose tissue enriched cluster, we found the mitochondrial creatine kinases, CK-MT1A/B and CK-MT2, abundantly and almost exclusively expressed in the brown adipose tissue samples (see Fig. 3a). The main enzymatic function of the mitochondrial creatine kinases is the conversion of creatine (Cr) to phospho-creatine (p-Cr) through the utilization of ATP. Thereby ADP is regenerated, a process in which creatine levels can regulate creatine kinase activity^22^. Additionally, the mitochondrial creatine kinases have a structural role by forming a functional complex with the voltage-dependent anion channel (VDAC) in the outer mitochondrial membrane (OMM) and the ADP/ATP-translocase, the phosphate transporter and the F1FO ATP-synthase (Complex V) in the inner mitochondrial membrane (IMM), which is termed the mitochondrial interactosome^23^. By juxtaposing these complexes, the mitochondrial creatine kinases enable the efficient flow of metabolites between these enzymes and facilitate the availability of high levels of ADP and phosphate as substrates for the ATP-synthase (see Fig. 3b). To characterize the functional importance of CK-MT1A/B and CK-MT2 in BAT, we analyzed the abundance of proteins involved in creatine metabolism as well as components of the mitochondrial interactosome. Arginase-1, an enzyme converting arginine into ornithine and thereby inhibiting creatine synthesis^24^, was significantly decreased in brown compared to white adipose tissue (see Supplementary Fig. S2). This decrease of Arginase-1 is indicative of a higher rate of creatine synthesis and CK activity. Furthermore, all components of the mitochondrial interactosome as well as most subunits of the ATP-synthase, were enriched in the human BAT compared to SAT (see Fig. 3c), suggesting a functional importance of coupled respiration in human BAT. Notably, the enrichment of ATP synthase (complex V) is in discordance with rodent data, as this protein complex is almost absent in interscapular brown adipose tissue^25^,^26^ and rather unchanged during the browning of subcutaneous white adipose tissue^27^. These findings demonstrate that human brown adipose tissue contains the protein complexes responsible for coupled respiration and their abundance is regulated in a concurrent manner to UCP1.

### *In vitro* differentiated hMADS cells utilize coupled and uncoupled respiration to a similar extend

**Figure 4:**
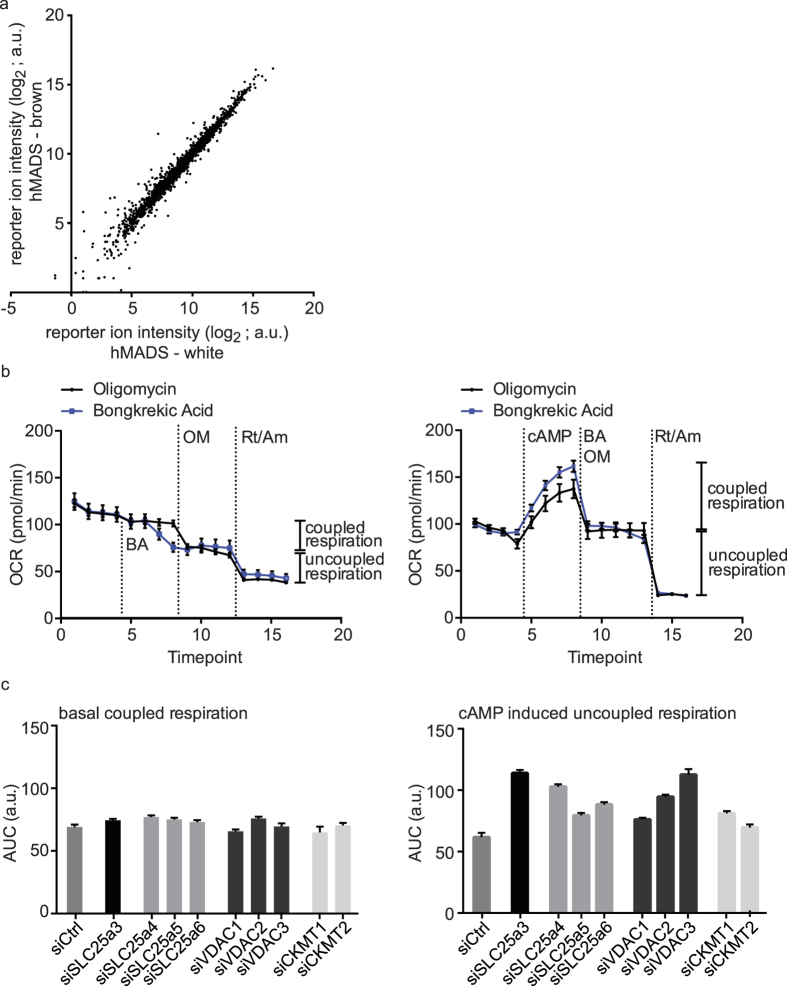
Bioenergetics profiling of *in vitro* differentiated brown hMADS cells demonstrates equal contribution of coupled and uncoupled respiration. (**a**) Scatter blot of white versus brown differentiated hMADS cells of the summed up protein intensities of all proteins quantified in the proteomic study. (**b**) OCR measurements in differentiated brown hMADS cells. Each timepoint represents a four minute measurement cycle, at the dotted lines the annotated compounds were injected. Data are presented as the mean +/− SEM (n = 6). Oligomycin was used for ATP synthase inhibition, bongkrekic acid for adenine nucleotide transporter inhibition, both were used to assess coupled respiration. cAMP was used to activate the brown adipocytes. Rotenone and antimycin A block OXPHOS complex I and III respectively and were used to assess non-mitochondrial respiration. Coupled respiration is mitochondrial respiration affected by oligomycin or bongkrekic acid; uncoupled respiration is mitochondrial respiration not affected by either inhibitor. (**c**) OCR measurements in differentiated brown hMADS cell treated with either control or siRNAs targeting components of the mitochondrial interactosome. Data are presented as area under curve (AUC) across 4 timepoints +/− SEM (n = 6) of coupled respiration or cAMP induced uncoupled respiration as defined in (**b**). Maximum coefficient of variation (CV) of the individual time-points contributing to the AUC was used for the calculation of SEM. Between plates, control siRNA mean values were normalized to the same absolute numbers. BA = bongkrekic acid; OM = oligomycin; Rt/Am = rotenone/antimycin A.

To compare our human tissue proteotype data with an established human white and brown adipocyte cell line model we next analyzed protein abundance in *in vitro* differentiated hMADS cells^19^. Interestingly, on a global proteome level, white and brown differentiated hMADS cells are remarkably similar (see Fig. 4a). Except for UCP1, we could not detect any differential abundance of the brown specific protein cluster, which we had identified using the human biopsy samples (see Supplementary Fig. S3). Although the globally different cellular composition within the cultured cells and clinical adipose tissue specimens makes a direct comparison inherently difficult, we showed that the median abundance of the proteins enriched in primary BAT is comparable in brown hMADS cells, therefore these cells can be considered an adequate model system (see Supplementary Fig. S3). Since the BAT enriched proteins involved in ATP-synthase coupled and uncoupled respiration rely on oxygen turnover to fulfill their function, we employed direct measurement of the oxygen consumption rate (OCR) in brown hMADS cells to elucidate to which extent they functionally contribute to overall cellular respiration. By using oligomycin, a complex V inhibitor, and bongkrekic acid, a specific inhibitor of the ADP/ATP translocase, we could show that in mature brown adipocytes in basal state, as well as when stimulated by cAMP, uncoupled and coupled respiration contribute to a similar degree to the OCR (see Fig. 4b). This confirmed the functional importance of ATP-synthase coupled respiration in human brown adipocytes, corroborating the results of the primary patient samples, which were based on protein abundance levels. Single siRNA mediated knock-down of the mitochondrial creatine kinases or other components of the mitochondrial interactosome showed no effect on coupled respiration, which can be explained by the high redundancy in the system^28^. Interestingly, however, each single knock-down led to an increase in uncoupled respiration (see Fig. 4c), suggesting that the coupled respiratory machinery can influence the rate of uncoupling. In conclusion, we observed that both the coupled and uncoupled mitochondrial energy utilization significantly contribute to the overall energy expenditure in brown hMADS cells and silencing experiments further support the interconnectedness of both pathways.

## Discussion

Since the discovery of active brown adipose tissue in adults, major research has focused on how to induce its formation and enhance its function. Functional assumptions are usually derived from rodent studies or transcriptome analyses of a limited amount of human biopsies, despite differences in vascularization^29^,^30^,^31^, localization^32^ and gene expression between human and mouse models are controversially discussed (reviewed in Sanchez-Gurmaches *et al*.^33^). Here we present the first proteomic scale study of human brown adipose tissue, in which we identify 318 proteins specifically enriched in BAT compared to SAT. The strong enrichment of all kinds of mitochondrial proteins in the brown compared to the white counterpart is in accordance with numerous rodent studies. These demonstrate, that mitochondria from brown adipose tissue compared to white are not just in bulk more abundant, but also show specific protein changes^26^. Of particular interest is the brown-specific enrichment of the CK-MT1A/B and CK-MT2 in a concurrent manner to UCP1. In a previous study of human brown adipose tissue, it has already been shown to express mitochondrial creatine kinase by immunohistochemistry^15^, here we expand this single protein evidence to the whole coupled respiratory machinery, including the F1FO-ATP synthase as well as proteins functionally interacting in the mitochondrial interactosome^23^. This supercomplex was initially described in heart muscle tissue with the mitochondrial creatine kinase as central component, to efficiently couple and regulate the flux of metabolites (ADP/ATP/phosphate; Cr/p-Cr) in order to ensure a high rate of productivity of the participating enzymes. The immediate importance of this protein complex in human BAT function, however, remains elusive. Kazak and colleagues describe in a recent study, that beige/brite adipocytes in mice utilize a creatine dependent ATP-synthase coupled futile cycle to expend energy and that in *in vitro* differentiated human cells, reduced creatine levels lower the oxygen consumption rate^34^. Our data show the presence of a similar system in primary human brown adipocytes. Despite the difficulties of quantitatively comparing the two studies, the raw data suggests on peptide-to-spectrum match (PSM) level that the mitochondrial creatine kinases are much more prominently expressed in the human compared to the rodent system (see Supplementary Table S3). Since it has been suggested, that human brown adipose tissue has several functions beyond heat production^35^, the coupled respiratory system could also serve homeostatic purposes in human brown adipocytes, like the regulation of reactive oxygen species levels^36^,^37^. However, a detailed functional analysis of the coupled respiratory system in human brown adipose tissue remains subject to further investigation.

In summary, we show that in human brown adipose tissue the protein machineries for uncoupled as well as coupled respiration are expressed in parallel. Although we could not detect inherently low abundant proteins, like putative BAT transcription factors, it has been shown, that a tissue can be functionally characterized by its abundantly expressed proteome^38^. Hence, we strongly believe, also apart from the specific findings outlined in the report, the presented proteotype will be of great utility to other studies elucidating the burning questions in human brown adipose tissue biology and its physiological functions.

### Experimental procedures

#### Chemicals

All chemicals, unless mentioned otherwise, were ordered from Sigma-Aldrich in at least HPLC grade purity.

#### Clinical study

The clinical study was approved by the Local Ethics Committee (University Hospital in Bratislava, Slovakia) and it conforms to the ethical guidelines of the 2000 Helsinki declaration. All study participants provided witnessed written informed consent prior entering the study. Deep neck and adjacent subcutaneous adipose tissue samples were obtained from the lower third of the neck by an experienced ENT surgeon from eleven patients (Supplementary Table S1). See Supplementary experimental procedures for further details.

#### Cell culture

Human multipotent adipose-derived stem (hMADS) cells were cultured as previously described^19^. For details see Supplementary experimental procedures.

#### Protein extraction and Western blot

Adipose tissue samples and *in vitro* differentiated adipocytes were homogenized in RIPA buffer (50 mM Tris pH 7.4, 150 mM NaCl, 2 mM EDTA, 1.0% Triton X100, 0.5% sodium deoxycholate, 0.1% SDS) supplemented with protease (Roche) and phosphatase (Thermo Fisher) inhibitor cocktails. Lysates were cleared by centrifugation at 12,000 g for 15 minutes at 40 °C. Protein concentration of the supernatants was determined by DC Protein Assay (Bio-Rad). Equal amount of proteins (40 μg) was separated on 12% SDS-polyacrylamide gel, transferred to a nitrocellulose membrane (PerkinElmer) and probed for UCP1 (1:1000, Thermo Fisher PA1-24894) and γ-tubulin (1:10,000 Sigma T6557). Signal of the HRP-conjugated secondary antibodies (Calbiochem) was visualized by the Image Quant system (GE Healthcare Life Sciences).

#### Sample preparation for MS analysis

Samples were prepared with the TMT10plex Isobaric Mass Tag Labeling Kit (Thermo Scientific, Lot number QD212963) and the Pierce High pH Reversed-Phase Peptide Fractionation Kit (Thermo Scientific) according to the manufacturer’s instructions. For details see Supplementary experimental procedures.

The labeling efficiency of this method was tested with the TMTzero label reagent (Thermo Scientific) followed by mass spectrometric analysis and determined to be higher than 98% (data not shown).

#### Mass Spectrometric data acquisition

Data acquisition was essentially performed as described^39^, for details see Supplementary experimental procedures.

#### Mass spectrometric data analysis

The acquired raw data was analyzed with Proteome Discoverer 2.1 (Thermo Scientific), for complete settings see Supplementary experimental procedures^41^.

For quantification, the normalized intensities on the protein level were averaged between the technical replicates. A protein was only deemed as consistently and reproducibly quantified, if a quantification value was obtained in both technical replicates in more than half of the individual samples.

#### Cellular respiration

Cellular respiration was measured with the extracellular flux analyzer XF96 (Seahorse Bioscience) according to manufacturer’s instructions. For details see Supplementary experimental procedures.

#### Statistical methods and graphical representations

Unsupervised k-means clustering was performed in Spotfire 3.2.2 (Tipco) on the protein level quantification patient data, with correlation similarity as distance measure and 9 clusters as target. Line similarity analysis was performed in Spotfire 3.2.2 (Tipco) based on UCP1 expression inside the BAT enriched cluster, with correlation similarity as distance measure, to obtain a ranking of target proteins. Ratio paired t-test statistics, coefficients of variation and areas under the curve were calculated in Prism 6 (GraphPad). Pathway and gene-set enrichments were calculated by Enrichr^40^. As graphical representations of the data, the heat maps were generated with Spotfire 3.2.2 (Tipco), all other graphs with Prism 6 (GraphPad).

### Data Availability

The mass spectrometry proteomics data have been deposited to the ProteomeXchange Consortium (http://proteomecentral.proteomexchange.org) via the PRIDE partner repository with the dataset identifier PXD003843.

## Additional Information

**How to cite this article**: Müller, S. *et al*. Proteomic Analysis of Human Brown Adipose Tissue Reveals Utilization of Coupled and Uncoupled Energy Expenditure Pathways. *Sci. Rep.* **6**, 30030; doi: 10.1038/srep30030 (2016).

## Supplementary Material

Supplementary Information

Supplementary Table S2
