# Supplementary material for: Proteomic Analysis of Human Brown Adipose Tissue Reveals Utilization of Coupled and Uncoupled Energy Expenditure Pathways
Source: Sci Rep. 2016 Jul 15;6:30030. doi: 10.1038/srep30030 (PMC4945940; doi:10.1038/srep30030)
Supplement: Supplementary Information [file srep30030-s1.pdf]

Proteomic Analysis of Human Brown Adipose Tissue Reveals Utilization of Coupled and Uncoupled Energy Expenditure Pathways

Sebastian Müller*, Miroslav Balaz*, Patrik Stefanicka, Lukas Varga, Ez-Zoubir Amri, Jozef Ukropec, Bernd Wollscheid**, Christian Wolfrum**

* Equal contribution

** Co-senior author

Supplementary Material

Supplementary Figure S1: Blood derived contaminants and common reference proteins indicate no bias towards SAT or BAT samples in the downstream analysis

(A) Summed up reporter ion intensities of serum derived proteins Albumin, Transferrin and Apolipoprotein B100
(B) Summed up reporter ion intensities of erythrocyte derived proteins Hemoglobin beta erythroctic isoforms of spectrin alpha and beta
(C) Summed up reporter ion intensities of reference proteins Actin, Tubulin α and β, heat shock protein 90 (HSP90) and 60S acidic ribosomal protein P0 (RPLP0)

Data was analyzed by ratio paired t testing of the SAT and BAT sample pairs (n=8); *p < 0.05; **p < 0.01; ***p < 0.001.

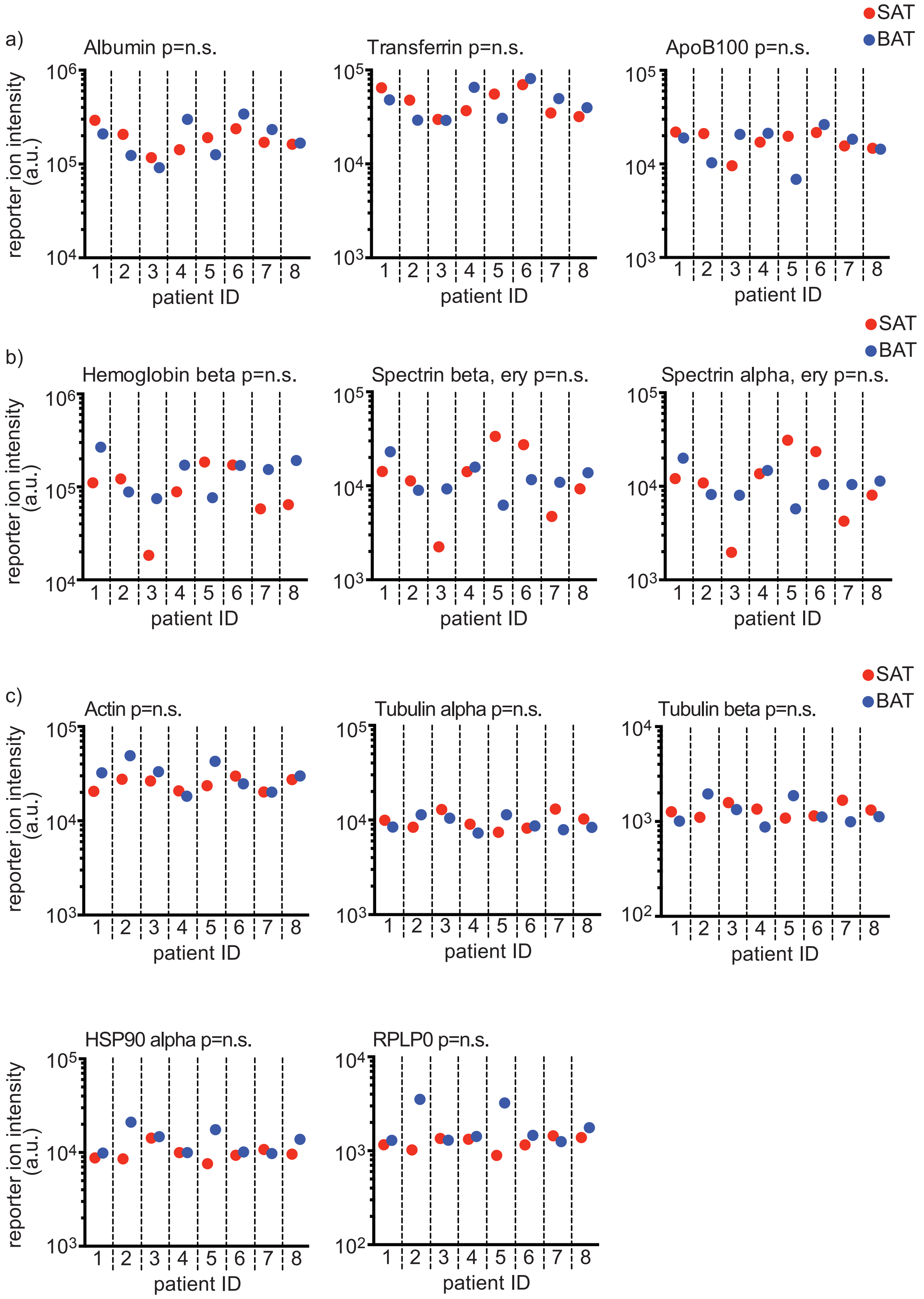

Supplementary Figure S2: Creatine metabolism in the SAT and BAT paired patient data

(A) Excerpt of the arginine and proline metabolism in the KEGG database surrounding the creatine pathway.
Proteins in red are quantified in our study, proteins in grey were not identified.
Blue arrows depict an enzymatic reaction, red dotted arrow an inhibitory interaction.
(B) Summed up reporter ion intensities for proteins involved in the creatine pathway, as indicated in (A)

Data was analyzed by ratio paired t testing of the SAT and BAT sample pairs (n=8); *$p < 0.05$; **$p < 0.01$; ***$p < 0.001$.

ALDH18A=Delta-1-pyrroline-5-carboxylate synthase; ARG=Arginase; CKB= cytosolic creatine kinase B-type
CKMT1/2=mitochondrial creatine kinase U-type and S-type; GATM=glycine amidinotransferase;
GAMT=guanidinoacetate N-methyltransferase; OAT=ornithine aminotransferase; PYCR=pyrroline-5-carboxylate reductase

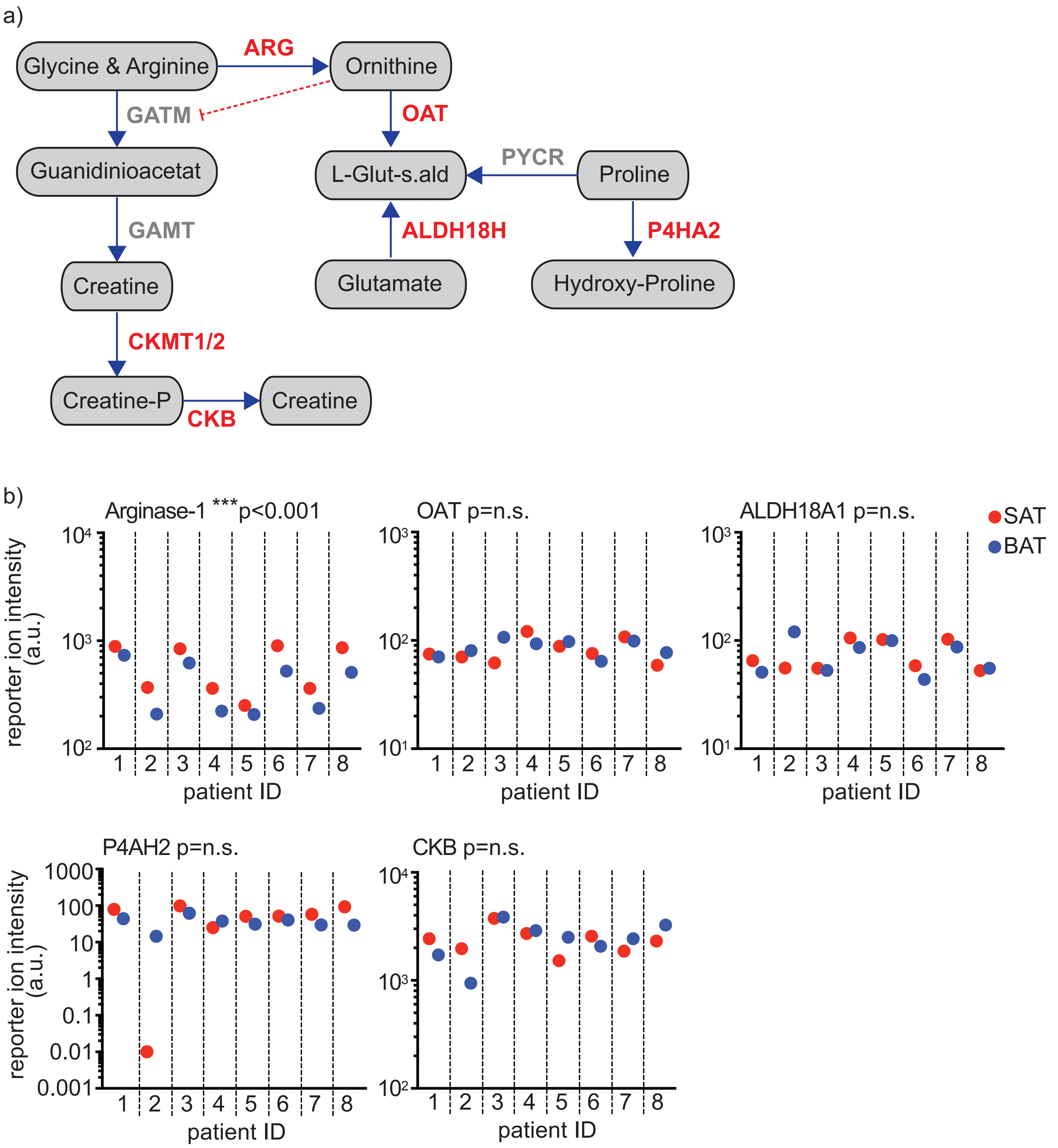

Supplementary Figure S3: White and brown in vitro differentiated hMADS cells analyzed on a whole proteome level

(A) Heat map as shown in figure 3, including here the hMADS cells data.
(B) Summed up reporter ion intensities for all 318 proteins in the BAT enriched cluster shown across paired patient samples and differentiated brown hMADS cells.
The median expression level is indicated by the horizontal line.

a)

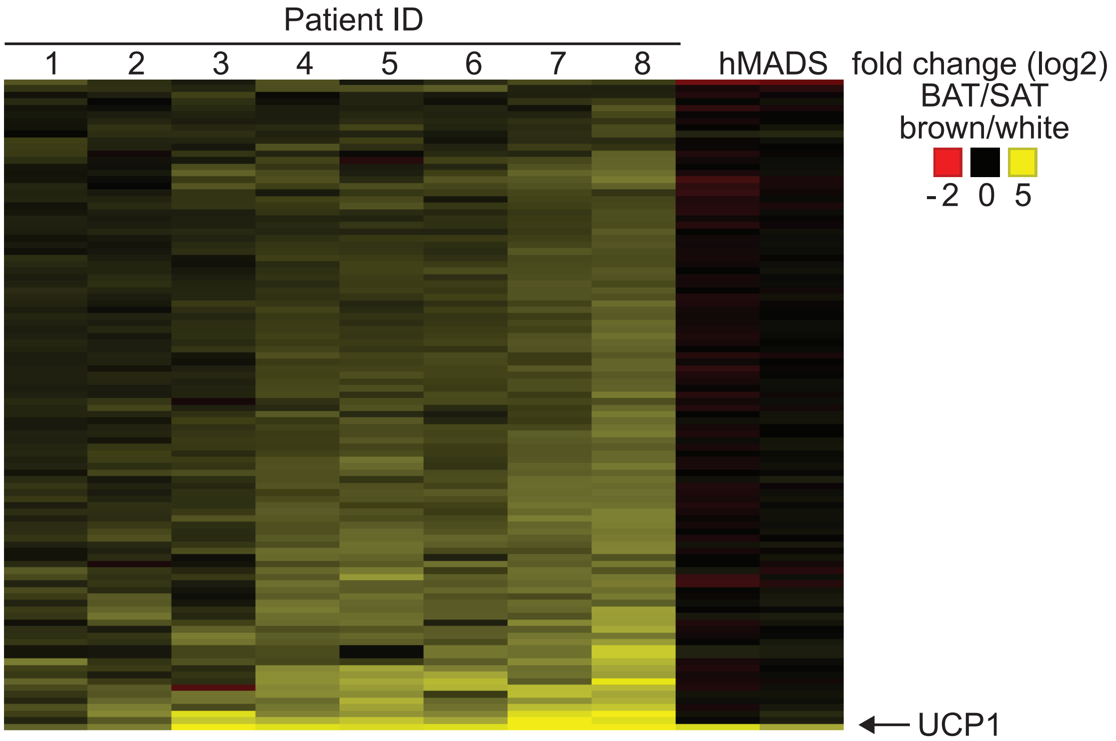

b)

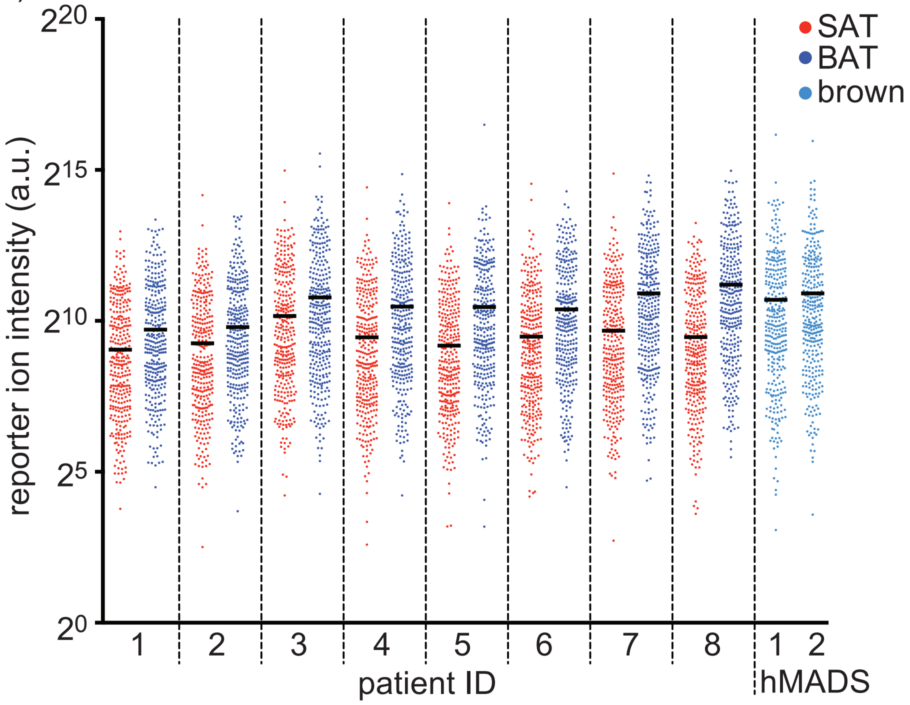

Supplementary Table S1: Patient cohort characteristics

	Patient 1	Patient 2	Patient 3	Patient 4
**Original Study ID**	BF24	BF5	BF19	BF7
**Age**	60	32	60	34
**Sex**	female	female	female	female
**BMI**	28.1	22.6	20.7	19.3
**height (cm)**	160	163	160	169
**weight (kg)**	72	60	53	55
**waist circumference (cm)**	88	74	77	68
**body fat (%)**	37.2	30.3	27.7	25.6
**diagnosis**	nodular goiter	nodular goiter	diffuse and nodular goiter	nodular goiter

	Patient 5	Patient 6	Patient 7	Patient 8
**Original Study ID**	BF12	BF13	BF10	BF14
**Age**	22	45	55	31
**Sex**	female	male	female	female
**BMI**	26.0	22.3	22.4	25.6
**height (cm)**	184	164	158	158
**weight (kg)**	88	60	56	64
**waist circumference (cm)**	86	60	80	93
**body fat (%)**	17.4	28.4	28.5	38.1
**diagnosis**	lateral (branchiogenic) cervical cyst	follicular adenoma (thyroid)	parathyroid adenoma, primary hyperparathyroidism	thyroid nodule

	Patient 9	Patient 10	Patient 11
**Original Study ID**	BF9	BF17	BF21
**Age**	26	29	62
**Sex**	female	male	female
**BMI**	25.9	25.7	24.8
**height (cm)**	169	187	162
**weight (kg)**	74	90	65
**waist circumference (cm)**	105	96	86
**body fat (%)**	30.3	19.7	36.1
**diagnosis**	toxic diffuse goiter	thyroid nodule	parathyroid adenoma, primary hyperparathyroidism

Supplementary Table S2: Results of the proteomic study

Identifications: Listed are all proteins identified with high confidence

Quantification: Listed are all proteins consistently quantified with the summed up TMT reporter ion intensities reported

HumanBAT enriched cluster: Listed are all proteins in the human BAT enriched cluster

Supplementary Table S3: Comparison of this study to Kazak et al.

Listed are the peptide-to-spectrum-matches (PSMs) of the study by Kazak et al. and this study as an approximate quantitative comparison.
Similar amount of PSMs are detected for the VDACs, as a reference mitochondrial protein.
UCP1 is detected with 3-fold more PSMs in the study by Kazak at al., while CK-MT1 with approximately 100-fold and CK-MT2 10-fold more PSMs in our study.

Excerpt of Kazak et al. (Supplementary Table 1, Related to Figure 1; Beige and brown fat mitochondrial protein quantification):

**Protein**	**Gene Symb**	**Description**	**Peptides**
sp|Q60932-2|VDAC1_MOUSE	Vdac1	Isoform Mt-VDAC1 of Voltage-dependent anion-selective channel protein 1 OS=	**119**
sp|Q60930|VDAC2_MOUSE	Vdac2	Voltage-dependent anion-selective channel protein 2 OS=Mus musculus GN=Vd	**94**
sp|Q60931|VDAC3_MOUSE	Vdac3	Voltage-dependent anion-selective channel protein 3 OS=Mus musculus GN=Vd	**46**
sp|P12242|UCP1_MOUSE	Ucp1	Mitochondrial brown fat uncoupling protein 1 OS=Mus musculus GN=Ucp1 PE=	**304**
sp|P30275|KCRU_MOUSE	Ckmt1	Creatine kinase U-type, mitochondrial OS=Mus musculus GN=Ckmt1 PE=1 SV=	**1**
sp|Q6P8J7|KCRS_MOUSE	Ckmt2	Creatine kinase S-type, mitochondrial OS=Mus musculus GN=Ckmt2 PE=1 SV=	**8**

Excerpt of this study (supplementary table 2; result of the proteomic study):

**Protein**	**Gene**	**Description**	**# PSMs**	**# Unique Peptides**
P21796	VDAC1	Voltage-dependent anion-selective channel protein 1 OS=Homo sapiens GN=VD	**137**	18
P45880	VDAC2	Voltage-dependent anion-selective channel protein 2 OS=Homo sapiens GN=VD	**89**	14
Q9Y277	VDAC3	Voltage-dependent anion-selective channel protein 3 OS=Homo sapiens GN=VD	**94**	12
P25874	UCP1	Mitochondrial brown fat uncoupling protein 1 OS=Homo sapiens GN=UCP1 PE=	**96**	15
P12532	CKMT1B; C	Creatine kinase U-type, mitochondrial OS=Homo sapiens GN=CKMT1A PE=1 S	**100**	12
P17540	CKMT2	Creatine kinase S-type, mitochondrial OS=Homo sapiens GN=CKMT2 PE=1 SV=	**131**	19

Supplementary experimental procedures

Chemicals

All chemicals, unless mentioned otherwise, were ordered from Sigma-Aldrich in at least HPLC grade purity.

Clinical study

The clinical study was approved by the Local Ethics Committee (University Hospital in Bratislava, Slovakia) and it conforms to the ethical guidelines of the 2000 Helsinki declaration. All study participants provided witnessed written informed consent prior entering the study. Deep neck and adjacent subcutaneous adipose tissue samples were obtained from the lower third of the neck by an experienced ENT surgeon from eleven middle-aged (41.5 ± 4.6 years) non-obese (BMI 23.9 ± 0.8 kg/m2; waist circumference 83.0 ± 3.9 cm; body fat 29.0 ± 2.0 %) individuals (2M/9F) during neck surgery under general anesthesia (i.e. thyroid surgery (n=10) or branchial cleft cyst surgery (n=1); see also **table S1**). The deep neck adipose tissue sample was taken from pre- and paravertebral space between common carotid and trachea in case of thyroid surgery and just laterally to carotid sheath in case of branchial cleft cyst surgery. In all cases, the surgical approach was sufficient to reach and sample the deep neck adipose tissue without any additional morbidity. Patients with malignant disease and subjects younger than 18 years were excluded from participation in the study. Adipose tissue samples were immediately cleaned from blood and connective tissue, and frozen in liquid nitrogen until further processing. Patients where the sample amount or quality was not sufficient for subsequent protein analysis were excluded from the study.

Cell culture

Human multipotent adipose-derived stem (hMADS) cells were cultured as previously [1]. Briefly, hMADS cells (passages 14 – 15) were grown in low glucose DMEM supplemented with 15mM HEPES, 10% FBS, 2mM L-glutamine, 1% Penicillin/Streptomycin and 2.5 ng/ml recombinant human FGF-2 (Peprotech). The medium was changed every other day and FGF-2 was omitted after cells reached confluence. Differentiation of 2-day post-confluent cells was induced (day 0) by adipogenic medium (DMEM/Ham’s F12 media containing 10 µg/ml Transferrin, 10nM insulin and 0.2 nM triiodothyronine) supplemented with 1 µM dexamethasone and 500 µM isobutyl methylxanthine (IBMX) and from day 2 to 9, cells were cultured in adipogenic medium containing 100nM rosiglitazone. Cells were kept in culture until day 18 in absence of rosiglitazone to obtain mature white adipocytes. To obtain brown adipocytes, cells were exposed to an additional rosiglitazone pulse between days 14 and 18. To knockdown candidate genes/proteins, 50nM siRNA pool (3 siRNAs per gene, see table below, Microsynth) was delivered into mature adipocytes (day 13) using Lipofectamine RNAiMAX (Invitrogen). After 24 hours, transfection medium was replaced by fresh adipogenic medium containing 100 nM rosiglitazone. Brown adipocytes were cultured until day 18 when cellular respiration was

determined or cells were harvested for protein analysis. Knockdown efficiency of this procedure was assessed by qRT-PCR and found to be between 78 % and 99 % on RNA level (data not shown).

siRNA Name	Sense Strand Sequence (Core Seq.) 5'-3'
h_Slc25a4_n1	GCAGUACAAAGGGAUCAUU
h_Slc25a4_n2	GCAGUUCUGGCGCUACUUU
h_Slc25a4_n3	CCAAUGUGCUGAGAGGCAU
h_Slc25a5_n1	GGGUUGACUUCCUAUCCAU
h_Slc25a5_n2	UCGUCAUCAGCUGGAUGAU
h_Slc25a5_n3	CCAAGAACACUCACAUCGU
h_Slc25a6_n1	GCCUGGUGAAGAUCACCAA
h_Slc25a6_n2	UCGUGUACCCGCUGGAUUU
h_Slc25a6_n3	GCAGGGCAUCAUCAUCUAC
h_Slc25a3_n1	GCAGGGACUCUGCAAGUUU
h_Slc25a3_n2	GCUAAGGUUCGAAUUCAAA
h_Slc25a3_n3	GCAGCUGGUUGUAACAUUU
h_VDAC1_n1	CCAGGGAUGUCUUCACCAA
h_VDAC1_n2	GGAGACCGCUGUCAAUCUU
h_VDAC1_n3	GGACUGGAAUUUCAAGCAU
h_VDAC2_n1	GCUGACAAGGAAUAACUUU
h_VDAC2_n2	GCUACACACUAAUGUCAAU
h_VDAC2_n3	GGGACAGAAUUUGGAGGAU
h_VDAC3_n1	GCAUCAGGCAACCUAGAAA
h_VDAC3_n2	GGUCUGUAACUAUGGACUU
h_VDAC3_n3	CCAAACUGUCACAGAAUAA
h_CKMT2_n1	GCUUCUCUGCUGUUUGCUA
h_CKMT2_n2	GCAACAAGGUGACACCCAA
h_CKMT2_n3	GCGAUUCUGUCGUGGACUA
h_CKMT1_n1	GCUUAUUGAUGACCACUUU
h_CKMT1_n2	CCAGAUGCUCGUGGAAUUU
h_CKMT1_n3	GGAGAGACUUAUCCAAGAA
Ctrl_n1	UGGUUUACAUGUCGACUAA
Ctrl_n2	UGGUUUACAUGUUUUCUGA
Ctrl_n3	UGGUUUACAUGUUUUCCUA

Protein extraction and Western blot

Adipose tissue samples and in vitro differentiated adipocytes were homogenized in RIPA buffer (50 mM Tris pH 7.4, 150 mM NaCl, 2 mM EDTA, 1.0 % Triton X100, 0.5 % sodium deoxycholate, 0.1 % SDS) supplemented with protease (Roche) and phosphatase (Thermo Fisher) inhibitor cocktails. Lysates were cleared by centrifugation at 12,000 g for 15 minutes at 40 °C. Protein concentration of the supernatants was determined by DC Protein Assay (Bio-Rad). Equal amount of proteins (40 µg) was separated on 12% SDS-polyacrylamide gel, transferred to a nitrocellulose membrane (PerkinElmer) and probed for UCP1 (1:1000, Thermo Fisher PA1-24894) and γ-tubulin (1:10,000 Sigma T6557). Signal of the HRP-conjugated secondary antibodies (Calbiochem) was visualized by the Image Quant system (GE Healthcare Life Sciences).

TMT labeling

TMT labeling was performed with the TMT10plex Isobaric Mass Tag Labeling Kit (Thermo Scientific, Lot number QD212963) according to the manufacturer's instructions. In detail, 40 µg of each adipose tissue protein extract were reduced with 10 mM Tris(2-carboxyethyl)phosphine (TCEP) at 55 °C for 60 min. Next, samples were alkylated with 20 mM Iodoacetamide at 24 °C for 30 minutes in the dark, before being precipitated by chloroform/methanol and washed with ice-cold methanol. Samples were re-solubilized in 100 mM triethyl ammonium bicarbonate (TEAB, Thermo Scientific), protein concentrations were determined with the Pierce BCA assay (Thermo Scientific) and protein amounts between the paired SAT and BAT samples readjusted to equal amounts. Proteins were digested with sequencing grade Trypsin (Promega) at a ratio of 1:40 (weight/weight) for 16 hours at 37 °C. The TMT labeling reagents were solubilized in anhydrous acetonitrile and 400 µg added to each patient / cell line sample according to the scheme in **Fig. 1b**. The reaction was stopped after 60 minutes at 24 °C with 5% hydroxylamine (Thermo Scientific) and the sets of samples mixed. The acetonitrile in the combined samples was removed with a speed-vac vacuum concentrator. The labeling efficiency of this method was tested beforehand with the TMTzero label reagent (Thermo Scientific) followed by mass spectrometric analysis and determined to be higher than 98 % (data not shown).

Sample pre-fractionation

Sample pre-fractionation was performed with the Pierce High pH Reversed-Phase Peptide Fractionation Kit (Thermo Scientific) according to the manufacturer's instructions. In detail, the storage solution of the C18 columns was removed (5000 x g for 2 min), the columns equilibrated with anhydrous acetonitrile (AcN) twice (5000 x g for 2 min) and conditioned with 0.1 % triethyamine (TEA; Thermo Scientific) there times (5000 x g for 2 min). The combined sets of samples were diluted to 300 µl with 0.1 % TEA and loaded on the columns (3000 x g for 2 min). The sample was washed once with 0.1 % TEA and once with 5 % AcN in 0.1 % TEA (3000 x g for 2 min each). Peptides were eluted stepwise with the following concentrations of AcN in 0.1 % TEA: 10 %, 12.5 %, 15 %, 17.5 %, 20 %, 22.5 %, 25 % and 50 % (300 µl; 3000 x g for 2 min each). All fractions were concentrated to dryness in a speed-vac vacuum concentrator.

Mass Spectrometric data acquisition

Peptides from each fraction were re-solubilized in 0.1% formic acid (FA) in a branson-type sonication-bath before being subjected to mass spectrometric analysis.

Each sample was separated by reversed-phase chromatography on a high pressure liquid chromatography (HPLC) column (75-µm inner diameter, New Objective) that was packed in-house with a 15-cm stationary phase (ReproSil-Pur C18-AQ, 1.9 µm) and connected to a nano-flow HPLC combined with an autosampler (EASY-nLC 1000; Thermo Scientific). The HPLC was coupled to an Orbitrap Fusion tribrid mass spectrometer (Thermo Scientific) equipped with a nanoelectrospray ion

source (Thermo Scientific). Buffer A was composed of 0.1 % FA in HPLC-grade water, Buffer B 0.1 % FA in HPLC-Grade AcN.

Data was acquired with modified settings, termed either TopN or TopSpeed.

TopN: Peptides were eluted in a continuous gradient with a flow rate of 300 nl per minute, starting at 2 % Buffer B, ramping to 8 % in 6 minutes, followed by an increase to 24 % over 64 minutes, followed by an increase to 28 % over 8 minutes, followed by an increase to 44 % Buffer B in 8 minutes and washing of the column in 80 % Buffer B. The mass spectrometer was operated in data-dependent acquisition (DDA) mode, consisting of a MS1 scan with the orbitrap as detector (Resolution: 120000; Scan range 400 – 1400 m/z; AGC target 2.0e5; maximum injection time: 100 ms). The 8 most intense precursor ions derived from a survey scan were fragmented via collision-induced-dissociation (CID) and analyzed as MS2 scan in the ion trap (Collision energy 35 %; AGC target 4.0e3; maximum injection time 150 ms, Scan Rate: rapid). As described [2] the 10 most intense ions of the MS2 were fragmented by higher-energy collisional dissociation (HCD) and analyzed in parallel in the orbitrap as MS3 scan (Resolution: 60000, Collision energy: 55%, AGC target: 5e4, maximum injection time 150 ms), to quantify the TMT reporter ions. The orbitrap and ion trap detectors operated in parallel.

TopSpeed: Peptides were eluted in a continuous gradient with a flow rate of 300 nl per minute, starting at 2 % Buffer B, ramping to 8 % in 6 minutes, followed by an increase to 24 % over 64 minutes, followed by an increase to 28 % over 8 minutes, followed by an increase to 44 % Buffer B in 8 minutes and washing of the column in 80 % Buffer B. The mass spectrometer was operated in DDA mode, consisting of a MS1 scan with the orbitrap as detector (Resolution: 120000; Scan range 400 – 1400 m/z; AGC target 2.0e5; maximum injection time: 100 ms). The most intense precursor ions were fragmented via CID and analyzed as MS2 scan in the ion trap (Collision energy 35 %; AGC target 1.0e4; maximum injection time 50 ms, Scan rate: turbo). As described [2] the 10 most intense ions of the MS2 were fragmented by HCD and analyzed in parallel in the orbitrap as MS3 scan (Resolution: 60000, Collision energy: 55 %, AGC target: 1e5, maximum injection time 50 ms), to quantify the TMT reporter ions. The orbitrap and ion trap detectors operated in parallel, the number of ions fragmented for MS2 and MS3 analyses was automatically optimized for a 3 second work-cycle.

Mass spectrometric data analysis

The acquired raw data was analyzed with Proteome Discoverer 2.1 (Thermo Scientific) en bloc. For the identification of the MS2 spectra, Sequest HT was utilized, with a 10 ppm precursor mass tolerance, 0.8 Da as fragment mass tolerance and as database, the Swissprot part of Uniprot for the species human was used (version 2016_01; 20145 entries). As dynamic modifications, oxidation (DeltaMass="15.99492") for all methionine residues was set and acetylation (DeltaMass="42.01057") for all protein N-termini. As static modifications were treated, the TMT-label (DeltaMass="229.16293") at the peptide N-terminus and all lysine residius and carbamidomethylation (DeltaMass="57.02146") at all cysteine residues. The fasle-dicovery-rate (FDR) for the peptide and consecutive protein assignments were performed by the included Percolator software package, based on a decoy database search and a strict cut-off of a 0.01 % FDR was applied on the protein level.

Reporter ion quantification was performed in the MS3 channel with a 20 ppm mass tolerance and corrected for the isotopic impurities provided for TMT lot QD212963. For the area calculations, the top 3 peptides were used and maximum fold change was set to 1000. No minimum vales were imputed. For the individual proteins, only unique and razor peptides were used for quantification. Overall the quantification was normalized by the total peptide amount in each channel. The data was enriched by multiple annotations, including gene ontology (GO) terms directly by Proteome Discoverer 2.1.
The data was filtered to contain only proteins with a FDR < 0.01 and being the "Master" or "Master-candidate" proteins in their respective protein group. The dataset was exported to Spotfire 3.2.2 (Tipco) for further processing.
For the quantification, the normalized intensities on the protein level were averaged between the technical replicates. A protein was only deemed as consistently and reproducibly quantified, if a quantification value was obtained in both technical replicates in more than half of the individual samples.

Cellular respiration

For measurement of cellular respiration, hMADS cells were differentiated and re-plated on collagen-coated 96-well Seahorse cell culture microplates at a density of 10.000 cells per well. On the day of experiment, adipogenic medium was replaced with unbuffered XF Assay Medium (pH 7.4, Seahorse Bioscience) supplemented with 25 mM glucose (Sigma), 2 mM sodium pyruvate (Invitrogen) and 2 mM GlutaMax (Invitrogen). The oxygen consumption rate (OCR) was measured using the Extracellular flux analyzer XF96 (Seahorse Bioscience). Test compounds were sequentially injected to obtain following concentrations: 1 µg/ml Oligomycin, 0.5 mM cAMP, 3 µM Rotenone with 2 µg/ml Antimycin A. To study the role of ATP/ADP translocase, 50 µM Bongkrekic acid was injected. OCR levels (pmol/min) were normalized to protein amount per well (µg protein) for AUC analysis. The data was analyzed with Prism 6 (GraphPad), including area under the curve calculation.

Statistical Methods

Unsupervised k-means clustering was performed in Spotfire 3.2.2 (Tipco) on the protein level quantification patient data, with correlation similarity as distance measure and 9 clusters as target. Line similarity analysis was performed in Spotfire 3.2.2 (Tipco) based on UCP1 expression inside the BAT enriched cluster, with correlation similarity as distance measure, to obtain a ranking of target proteins. Ratio paired t-test statistics and coefficients of variation were calculated in Prism 6 (GraphPad). Pathway and gene-set enrichments were calculated by Enrichr [4]. As graphical representations of the data, the heat maps were generated with Spotfire 3.2.2 (Tipco), all other graphs with Prism 6 (GraphPad).

References for supplementary experimental procedures

1 Elabd, C. *et al.* Human multipotent adipose-derived stem cells differentiate into functional brown adipocytes. *Stem Cells* **27**, 2753-2760, doi:10.1002/stem.200 (2009).
2 McAlister, G. C. *et al.* MultiNotch MS3 enables accurate, sensitive, and multiplexed detection of differential expression across cancer cell line proteomes. *Anal Chem* **86**, 7150-7158, doi:10.1021/ac502040v (2014).
3 Vizcaino, J. A. *et al.* ProteomeXchange provides globally coordinated proteomics data submission and dissemination. *Nat Biotechnol* **32**, 223-226, doi:10.1038/nbt.2839 (2014).
4 Chen, E. Y. *et al.* Enrichr: interactive and collaborative HTML5 gene list enrichment analysis tool. *BMC Bioinformatics* **14**, 128, doi:10.1186/1471-2105-14-128 (2013).
